# Neutrophil Extracellular Traps in Asthma: Friends or Foes?

**DOI:** 10.3390/cells11213521

**Published:** 2022-11-07

**Authors:** Remo Poto, Mohamed Shamji, Gianni Marone, Stephen R. Durham, Guy W. Scadding, Gilda Varricchi

**Affiliations:** 1 Department of Translational Medical Sciences, University of Naples Federico II, 80131 Naples, Italy; 2 Center for Basic and Clinical Immunology Research (CISI), University of Naples Federico II, 80131 Naples, Italy; 3 World Allergy Organization (WAO) Center of Excellence, 80131 Naples, Italy; 4 Immunomodulation and Tolerance Group, Allergy and Clinical Immunology, Inflammation, Repair, Development, National Heart and Lung Institute, Imperial College, London SW7 2AZ, UK; 5 Imperial College NIHR Biomedical Research Centre, Asthma UK Centre in Allergic Mechanisms of Asthma, London SW7 2AZ, UK; 6 Institute of Experimental Endocrinology and Oncology (IEOS), National Research Council (CNR), 80131 Naples, Italy; 7 Allergy and Clinical Immunology, National Heart and Lung Institute, Imperial College, London SW3 6LY, UK

**Keywords:** asthma, inflammation, neutrophils, neutrophil extracellular traps, NETs, PMN, TSLP, VEGF

## Abstract

Asthma is a chronic inflammatory disease characterized by variable airflow limitation and airway hyperresponsiveness. A plethora of immune and structural cells are involved in asthma pathogenesis. The roles of neutrophils and their mediators in different asthma phenotypes are largely unknown. Neutrophil extracellular traps (NETs) are net-like structures composed of DNA scaffolds, histones and granular proteins released by activated neutrophils. NETs were originally described as a process to entrap and kill a variety of microorganisms. NET formation can be achieved through a cell-death process, termed NETosis, or in association with the release of DNA from viable neutrophils. NETs can also promote the resolution of inflammation by degrading cytokines and chemokines. NETs have been implicated in the pathogenesis of various non-infectious conditions, including autoimmunity, cancer and even allergic disorders. Putative surrogate NET biomarkers (e.g., double-strand DNA (dsDNA), myeloperoxidase-DNA (MPO-DNA), and citrullinated histone H3 (CitH3)) have been found in different sites/fluids of patients with asthma. Targeting NETs has been proposed as a therapeutic strategy in several diseases. However, different NETs and NET components may have alternate, even opposite, consequences on inflammation. Here we review recent findings emphasizing the pathogenic and therapeutic potential of NETs in asthma.

## 1. Introduction

Asthma is a chronic inflammatory airway disease characterized by variable airflow limitation and airway hyperresponsiveness [1,2]. Asthma can be classified into two subgroups based on the type of inflammation: T (type) 2-high and T2-low [3]. T2-high asthma is the most frequent endophenotype, characterized by the activation of T2 helper cells (Th2), T2 innate lymphoid cells (ILC2), mast cells, basophils, and eosinophils, as well as the production of T2 cytokines (e.g., IL-3, IL-4, IL-5, and IL-13) [4,5,6]. In T2-high and T2-low asthma, several environmental (e.g., respiratory viruses, bacteria, fungi and cigarette smoke) and endogenous stimuli (e.g., cytokines and chemokines) can induce the release of epithelium-derived alarmins (i.e., thymic stromal lymphopoietin (TSLP), IL-33, and IL-25), which activate both innate and adaptive immune cells [4,7]. T2-low asthma biomarkers are currently lacking and represent an unmet need. Thus, T2-low asthma has a diagnosis of exclusion identified/characterized by the absolute and/or relative absence of T2 biomarkers [8].

Neutrophils, also called polymorphonuclear leukocytes (PMNs), are the first line of defense in fighting infections and maintaining tissue homeostasis [9]. PMNs account for approximately 65% of all leukocytes in humans and 10–20% in mice [10,11]. It has been estimated that humans produce ≅1 billion neutrophils per kilogram of body weight every day, and this may increase to 10 billion during inflammation [12]. Among their primary functions are degranulation, phagocytosis, and neutrophil extracellular trap (NET) release [13,14].

NETs were originally described as a defense mechanism to entrap and kill invading microorganisms [15]. During the last decades, NETs have been implicated in various conditions such as cancer [16,17,18,19], cardiovascular diseases [20,21,22], and autoimmune [19,23,24,25] and respiratory disorders [26]. A growing body of literature has emerged exploring the relationship between NETs and chronic inflammatory airway diseases, including asthma [27,28,29] and chronic obstructive pulmonary disease (COPD) [30]. In this review, we provide an overview of recent progress in understanding the formation of different types of NETs, the relationship between neutrophils and NETs in asthma, and the biomarkers associated with NETs, as well as a discussion of the possible therapeutic implications of NET inhibition in asthma.

## 2. Neutrophils in Inflammation

Neutrophils are fundamental actors in the host’s immune response [9]. Circulating PMNs are recruited to the site of inflammation, where they release a wide range of preformed and de novo synthesized mediators including several granular enzymes (e.g., neutrophil elastase (NE), myeloperoxidase (MPO), proteinase 3 (PR3), pentraxin 3 (PTX3)), lipid mediators (leukotriene B_4_), and reactive oxygen species (ROS) [31,32]. Beyond killing by degranulation and phagocytosis [33], neutrophils can use their chromatin to trap and kill microbes [12,15].

Neutrophils play an important role in the modulation of both innate and adaptive immune responses through their ability to modulate the behavior of several immune cells. These cells can act as antigen-presenting cells (APCs) [34,35,36], drive the recruitment, maturation and activation of macrophages and dendritic cells (DCs) [37,38,39,40], and release activating signals that contribute to the recruitment, activation, and differentiation of T cells [41] and B cells [42]. Therefore, neutrophils play a role in orchestrating inflammatory response [43]. At the site of inflammation, they are involved in complex and bidirectional interactions with other immune and stromal cells through the release of soluble mediators or direct cell-to-cell contact [44]. Furthermore, neutrophils seem to have more transcriptional activity than initially thought.

There is compelling evidence that human [45,46,47,48] and mouse [46,49,50,51] neutrophils express high levels of heterogeneity. Several neutrophil phenotypes have been identified in different experimental models and appear to display distinct functional properties. For example, neutrophils could undergo polarization toward antitumoral (N1) and protumoral (N2) phenotypes which differ in morphology and function [49,51,52]. There is also evidence that aged neutrophils express a certain degree of heterogeneity driven by the microbiota via toll-like receptor (TLR) activation [53].

Neutrophils can facilitate the resolution of inflammation through the release of pro-resolving lipid mediators [54,55], anti-inflammatory cytokines, such as IL-10 [56], and decoy receptors that act as scavengers of chemokines and cytokines [57,58,59,60].

Therefore, neutrophils should not be viewed as solely destructive cells, but rather their pleiotropic properties, both beneficial and detrimental, should be considered in the context of infections and environmental triggers that could also modulate the neutrophil decision-making algorithm.

## 3. NET Formation

NETs are net-like structures composed of DNA scaffolds associated with histones and several cytoplasmic granular proteins. Several immunologic and non-immunologic stimuli, including various bacteria [15], fungi [61] and viruses [62,63] activate human neutrophils to release NETs. Epithelial-derived alarmins (e.g., IL-33) can also activate neutrophils to produce ROS and NETs [64,65]. Similarly, TSLP stimulates eosinophils to produce other NET-like structures, known as eosinophil extracellular traps (EETs) [66].

**Figure 1:**
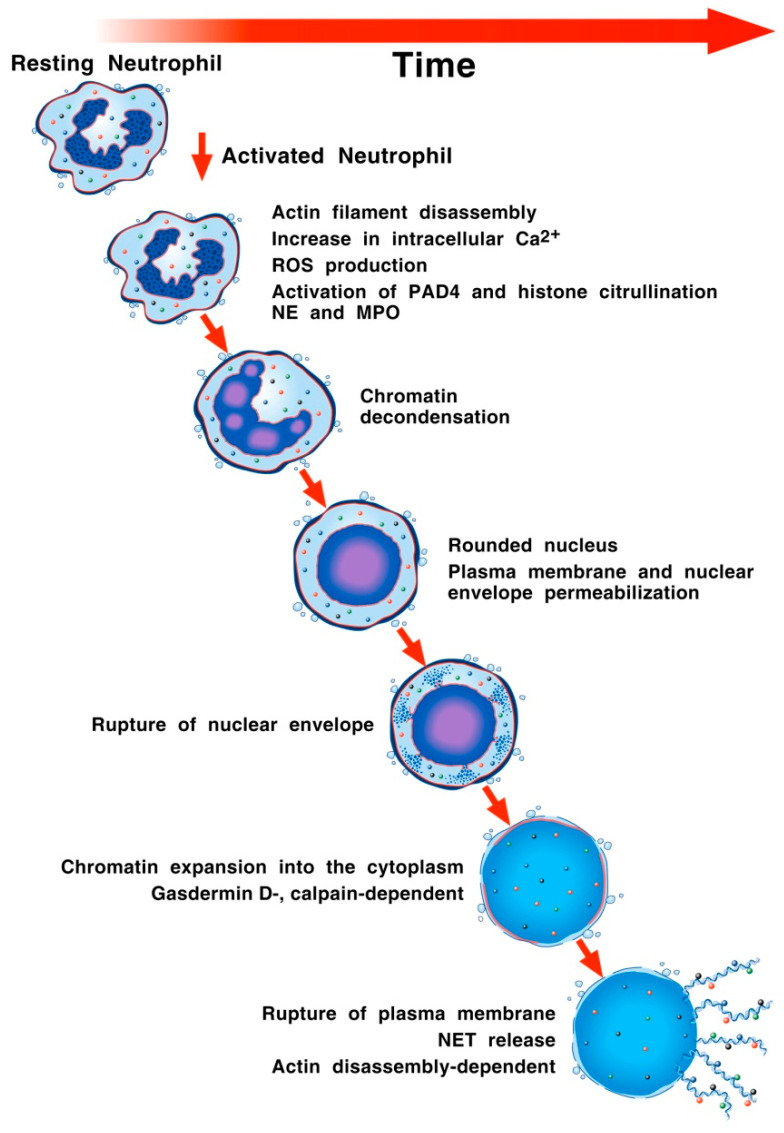
Schematic representation of biochemical and cellular events driving lytic NET formation. A multitude of extracellular pathogens, including viruses and bacteria, can induce lytic NET formation, also called NETosis [18,75]. Activation of neutrophils induces actin filament disassembly, an increase in intracellular Ca^2+^ and ROS production. Neutrophil elastase (NE) [68] and myeloperoxidase (MPO) [76] contribute to nuclear membrane permeabilization and unfolding of chromatin. Peptidyl arginine deiminase 4 (PAD4), mainly expressed in the nucleus of granulocytes, mediates citrullination of the nucleosome histones leading to chromatin decondensation [77]. These events favor nuclear rounding and nuclear envelope permeabilization. The following event is the chromatin swelling into the cytoplasm and the activation of gasdermin D [78]. A final step is the plasma membrane rupture and NET release [79].

NET formation was initially proposed to be a process of programmed cell death initiated by the activation of nicotinamide adenine dinucleotide phosphate (NADPH) oxidase followed by chromatin decondensation, breakdown of the nuclear membrane, and mixing of the chromatin with granular proteins [67]. This process is mediated by MPO, NE, and peptidyl arginine deiminase (PAD)-4 [68,69], which leads to the extrusion of DNA scaffolds decorated with citrullinated histones and granular proteins [70]. Further research has questioned whether all steps in this pathway are essential for the development of NETosis. Indeed, human saliva can induce NETosis independently of NADPH oxidase and NE [71]. Therefore, NET extrusion can be NADPH enzyme-dependent or independent, and the formation process can be divided into NETosis involving cell death (suicidal NETosis) and NET formation in which neutrophils remain alive (vital NET formation) [18,72,73,74]. Figure 1 schematically illustrates the biochemical events and morphological changes associated with NETosis following the activation of human neutrophils.

**Figure 2:**
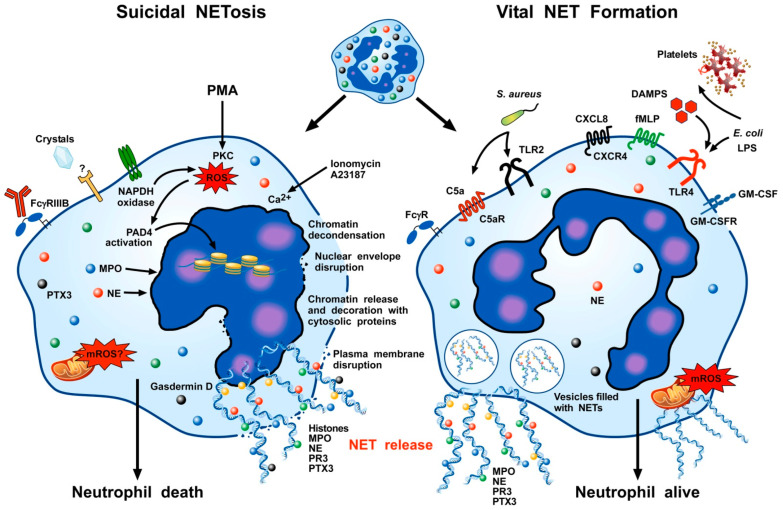
The most potent inducer of NETosis in vitro is phorbol 12-myristate 13-acetate (PMA), which activates protein kinase C (PKC) in human neutrophils. [15]. Several other stimuli such as agonists of FcγRIIIB [86], crystals [86,87], and the Ca^2+^ ionophore A23187 or ionomycin [88] can induce suicidal NETosis. NET formation induced by PMA requires NADPH (nicotinamide adenine dinucleotide phosphate) oxidase activity, but the requirement for this enzymatic activity differs depending on the stimulus [89]. Neutrophil elastase (NE) translocates from the cytoplasmic granules to the nucleus and activates its proteolytic activity in a myeloperoxidase (MPO)-dependent manner [90]. In the nucleus, NE degrades specific histones promoting chromatin decondensation [68]. Peptidyl arginine deiminase 4 (PAD4), which converts arginine residues into citrulline [91], catalyzes histone citrullination [69]. This results in a loss of positive charges on arginine residues in histones, which loosen the forces between DNA and histones and contributes to chromatin decondensation [92]. After nuclear envelope disruption, the chromatin comes into contact with several cytoplasmic granules (e.g., NE, MPO, PR3 (proteinase 3), PTX3 (pentraxin 3)) [12]. The rupture of the plasma membrane and the consequent NET release are mediated by gasdermin D [78,93,94]. In vital NET formation, the cell remains intact and normal cellular functions of neutrophils, such as chemotaxis and phagocytosis, can still be carried out [18,72,73,80,95]. This process is biochemically distinct from suicidal NETosis. A variety of stimuli such as bacterial products [73,95], GM-CSF and C5a [72,96], immune complexes [81,96,97], lipopolysaccharide (LPS) [80,98,99], and conditioned media of cancer cells [18] can rapidly (within 30 min) induce vital NET formation. Vital NET formation occurs without nuclear membrane disruption and has been reported with DNA leaving the cytoplasm in vesicles [73,80,95]. DNA becomes decorated with granule proteins (e.g., NE, MPO, PR3) [72,96]. Mitochondria can mediate mitochondrial ROS (mROS) formation [18,97]. (DAMP (damage-associated molecular pattern); *E. coli*, *Escherichia coli*; *S. aureus*, *Staphylococcus aureus*).

The release of extracellular traps can also occur without cell death [18,72,73,74,80,81]. NETs released during vital NET formation are composed of nuclear and mitochondrial DNA (mtDNA) and can be formed independently of NADH oxidase or ROS. In contrast to suicidal NETosis, vital NET formation is a rapid process occurring within 60 min and without affecting neutrophil viability [66,72,73,82]. Figure 2 schematically illustrates the fundamental mechanisms of suicidal neutrophils cytolysis (also termed NETosis) and of vital NET formation.

NET clearance is an endocytic process promoted by macrophages and DCs [83,84]. These cells are able to degrade NETs by interplaying with extracellular and secreted DNases followed by intracellular uptake [85].

## 4. Neutrophils in Asthma

The role of neutrophils in asthma pathobiology is complex, multifactorial, and largely unknown [2,100]. Earlier studies showed that circulating neutrophils are activated in patients with asthma during exercise [101] and following allergen inhalation challenge [102] as shown by enhanced surface complement receptor expression. Recently, we demonstrated that highly purified neutrophils isolated from the peripheral blood of patients with asthma and healthy individuals produced ROS in response to lipopolysaccharide (LPS) and N-Formyl-Met-Leu-Phe (fMLP), which activate distinct surface receptors (TLR4 and FPR, respectively) [19,103,104]. ROS production was lower in PMNs from patients with asthma compared to healthy subjects. Moreover, the mobilization of two neutrophil surface markers, CD11b and CD62L, induced by LPS and fMLP was more prominent in cells of healthy individuals compared to cells obtained from patients with asthma. The latter results are compatible with the hypothesis that circulating neutrophils from patients with asthma are chronically stimulated by numerous stimuli in vivo, which render these cells hyporesponsive to stimulation in vitro [103].

Neutrophils contain several preformed granular components that can be rapidly released [105]. Plasma concentrations of the neutrophil-derived mediators MPO, matrix metallopeptidase 9 (MMP-9) and CXCL8 were augmented in patients with asthma compared to healthy subjects [103], which suggests that PMNs are activated in vivo. MPO is a cationic enzyme located in primary azurophilic granules that catalyzes ROS production [106] and is involved in NET formation [19,68]. MPO also stabilizes NET structure by promoting the incorporation of chlorinated polyamines and the formation of covalent crosslinks [107]. Circulating levels [103] and sputum concentrations of MPO [108] were increased in patients with asthma compared to healthy individuals and correlated with extracellular DNA, a putative NET biomarker [108].

MMP-9, present within tertiary granules of neutrophils, has been detected in the bronchoalveolar lavage (BAL) of patients with asthma [109] and is associated with neutrophil counts [110]. In a murine model of asthma, MMP-9 knockout mice exhibited a reduced level of immune cell infiltration as well as decreased bronchial hyperresponsiveness compared to wild-type mice [111]. MMP-9 levels were increased in the BAL and sputum of patients with asthma after allergen challenge [111,112]. Moreover, neutrophil-derived MMP-9 was elevated in patients with severe asthma [110]. Higher levels of CXCL8 in circulation [103,113,114,115,116], sputum [117], and BAL fluid [16,118] were found in patients with asthma compared to healthy subjects.

The S100 family of calcium binding proteins is localized in the cytoplasm and/or nucleus of a wide range of cells, including neutrophils [119]. The calcium-binding protein A9 (S100A9), also known as calgranulin B, is secreted by activated neutrophils and can induce NET formation [120]. Increased levels of S100A9 were observed in the serum and sputum of patients with neutrophilic asthma, especially in those with uncontrolled disease [121,122]. S100A12, known as calgranulin C and secreted upon neutrophil activation, has also been associated with mast cell activation and allergic responses [123]. Surprisingly, S100A12 has been reported to reduce airway smooth muscle cells and dampen airway inflammation and hyperresponsiveness in a murine model of allergic lung inflammation [124].

Angiogenesis, the formation of new blood vessels, and lymphangiogenesis, the formation of new lymphatic vessels, occur in different inflammatory disorders including asthma [125,126,127] and cancer [52]. Vascular endothelial growth factors (VEGFs) are the most specific growth factors for blood and lymphatic endothelial cells [128,129]. Angiopoietins (ANGPTs) are members of another family of promoters of embryonic and post-natal neovascularization [130]. Human neutrophils can release several angiogenic factors (e.g., VEGF-A, ANGPT1) [52,131].

## 5. NETs in Asthma

NETs can be induced by several immunologic and non-immunologic stimuli. IL-33 [64,65], CXCL8 [18,132,133,134], IL-17 [135], C3a/C5a [72,136,137], IL-1β [99,108] and LPS [18,72,80,138], associated with asthma pathophysiology [108,139], are important inducers of NET formation. Moreover, several bacterial products (e.g., fMLP) [136,140,141], rhinovirus and influenza virus can induce NET formation [139,142].

The airway epithelium is the first line of defense for the lungs, which detects inhaled environmental threats such as allergens, superallergens, bacterial, fungal and viral products, and smoke extracts [143]. In patients with asthma, environmental and endogenous insults damage the epithelial cells, representing the first immunologic event in bronchial asthma [144,145]. Epithelial-derived cytokines [i.e., TSLP, IL-33, IL-25] act as upstream cytokines in asthma pathobiology [4,144,145,146,147]. NETs disrupt bronchial epithelial tight junctions, leading to intracellular component spillover [148]. NETs directly act on bronchial epithelial cells (BECs) to secrete inflammatory mediators that increase airway inflammation and provoke respiratory symptoms [108,149]. NET components, such as high-mobility group box 1 protein (HMBG1), can activate BECs to release several mediators involved in asthma pathogenesis, including TSLP, TNF-α, MMP-9, and VEGF [150]. Therefore, NETs could contribute to asthma pathobiology and exacerbations through the loss of integrity of the bronchial epithelium and the release of “upstream cytokines’’ such as alarmins (e.g., TSLP, IL-33).

In a murine model of asthma, Toussaint et al. found increased neutrophil numbers and NET formation on day 1 following rhinovirus exposure [139]. Akk and collaborators found that NET formation peaked two days following infection with Sendai virus, and that NETs played a critical role in the early phases of the immune response by recruiting and activating CD4^+^ and CD8^+^ T cells, increasing TNF-α and IL-6, and causing airway hyperresponsiveness [151]. NETs stimulated the presentation of antigens by DCs promoting a Th2 inflammatory response in a murine asthma model induced by LPS and house dust mites (HDM), as suggested by increased expression of T2 cytokines, eosinophil infiltration, mucus hypersecretion, and airway hyperresponsiveness (AHR) [152].

Recently, NETs have been implicated in an experimental model of air pollutant-induced asthma [153]. Diesel exhaust particles (DEPs) induced the release of adenosine triphosphate (ATP), promoting the expression of SiglecF on neutrophils. SiglecF^+^ PMNs produced increased NETs, cysteinyl leukotrienes (CysLTs) and ROS. Interestingly, a dual inhibitor of CysLTs and NETs reduced DEP-mediated asthma exacerbations [153]. The authors also found Siglec8^+^ (which corresponds to murine SiglecF^+^) PMNs in the sputum of patients with asthma-COPD overlap (ACO). Collectively these results indicate that early infiltration of neutrophils and the formation of NETs appear to be important factors in the initiation and development of experimental models of asthma.

NETs were identified in bronchial biopsy specimens of patients with allergic asthma [154]. NETs have also been proposed as biomarkers of asthma severity. The levels of extracellular DNA (eDNA), a NET component, were increased in sputum samples from patients with severe asthma compared to those with mild or moderate disease [27]. Plasma concentrations of dsDNA were increased in children with mild persistent asthma compared to healthy controls [155].

IL-4 and IL-13, the prototypic type 2 cytokines, signal through the activation of type 1 and type 2 IL-4Rs on human neutrophils [156]. IL-4 and IL-13 inhibited both phorbol 12-myristate 13-acetate (PMA)-induced NETosis and CXCL8-mediated chemotaxis of neutrophils. Moreover, NET formation induced by PMA in neutrophils from allergic subjects was reduced compared to healthy donors [156]. Finally, sera from allergic subjects inhibited PMA-induced NET formation and chemotaxis of healthy human neutrophils. Thus, it appears that NETs may have both protective and harmful roles in asthma pathogenesis. Moreover, these data indicate that neutrophils stimulated by Th2-like cytokines enter a state of activation or differentiation that differs from that of neutrophils from healthy subjects. Notably, PMA is a non-physiological stimulus recognized to induce NETosis. It would be interesting to evaluate whether similar phenomena are observed using stimuli relevant to the pathobiology of asthma.

Allergen immunotherapy (AIT) is a disease-modifying treatment for allergic rhinitis, allergic asthma, and Hymenoptera venom allergy [157,158]. A role for neutrophils in allergen processing and presentation to T cells has recently been demonstrated [159]. Recently, it has been demonstrated that aluminum hydroxide (alum) and monophosphoryl-lipid A (MPLA), traditional AIT adjuvants, can trigger the release of NETs from human neutrophils [160]. Moreover, vaccine formulations containing alum or MPLA can induce strong NET formation. Finally, NETs and alum can synergistically enhance the expression of molecules (i.e., CD80, CD86 and CD83) implicated in antigen presentation by peripheral blood monocytes [160]. Considering the dual effects of NETs (i.e., proinflammatory and anti-inflammatory), it will be interesting to evaluate the presence and role of NETs during AIT in patients with allergic disorders.

## 6. The Anti-Inflammatory Role of NETs

NETs are fundamental components of the innate immune system and play a key role in capturing and/or killing bacteria [15], viruses [161], fungi [162] and parasites [163]. Recent evidence suggests that NETs, under certain circumstances, can also have an anti-inflammatory role [9] and can modulate acute and chronic inflammation [164]. NETs have been shown to form barriers to prevent the spread of infections [165,166,167]. NETs and aggregated NETs (aggNETs) can promote the resolution of neutrophilic inflammation by degrading cytokines and chemokines, thus disrupting neutrophil recruitment and activation [168]. NETs and aggNETs also orchestrate the resolution of sterile crystal-mediated (e.g., monosodium urate, calcium pyrophosphate) inflammation [169]. Reduced NETosis leads to more severe disease in murine models of systemic lupus erythematosus (SLE) [170] and gout [168]. Degradation of NETs by DNase leads to a reduction of their antimicrobial properties [15]. NE decays virulence factors produced by Gram-negative bacteria [15]; whereas, serine proteases penetrate and destroy the bacterial membrane [171]. PTX3, a NET component, exerts antifungal activity [172], protects against extracellular histone-mediated cytotoxicity and mitigates the detrimental effect of NETs [172]. Furthermore, NETs can inhibit GM-CSF/IL-4-induced differentiation of monocytes into DCs in vitro, resulting in an alternatively activated macrophage phenotype [173]. This subset of macrophages is important in resolving and preventing chronic inflammation. Additionally, aggNETs prevent inflammation on the human neutrophil-rich ocular surface [10]. Collectively, these studies suggest that, under certain circumstances, NETs may provide platforms for degrading proinflammatory mediators that would otherwise drive inflammation.

**Figure 3:**
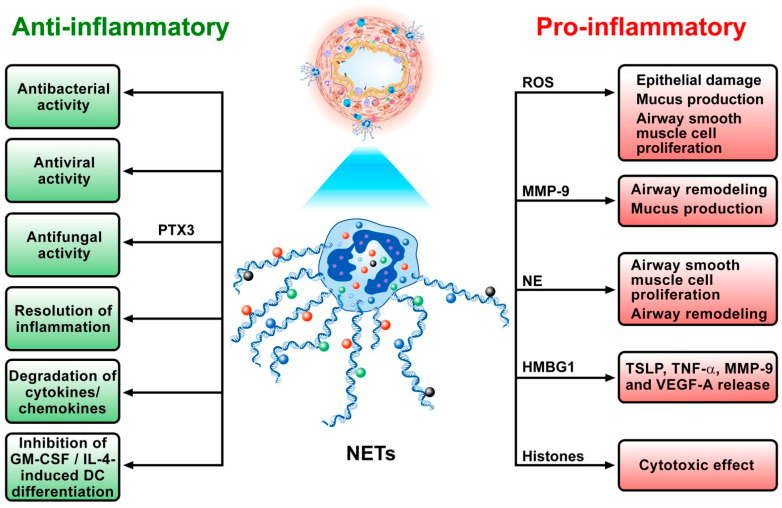
Schematic representation of the pleiotropic and potentially conflicting roles of NETs in human and experimental models of asthma. NETs and their components can exert a pathogenic role in asthma. In particular, they can contribute to epithelial and endothelial damage mediated by ROS, MMP-9 or NE [174]. ROS and NE can also favor airway smooth muscle cell proliferation [175]. MMP-9 contributes to airway edema and remodeling [112] and, together with ROS, stimulates mucus production [176]. High-mobility group box 1 protein (HMBG1) induces the release of TSLP, TNF-α, MMP-9 and VEGF-A from bronchial epithelial cells [150]. Histones exert a cytotoxic effect [177], and MMP-9 and NE participate in airway remodeling of the extracellular matrix (ECM) [178]. In an experimental model, NETs promoted rhinovirus-induced type 2 asthma exacerbations [139]. Neutrophil cytoplasts induced T_H_17 differentiation in a non-type 2 model of asthma [179]. NETs amplify neutrophil recruitment in a model of neutrophilic asthma [180]. NETs promoted neutrophil-associated asthma through the activation of macrophages [181]. In experimental models of asthma, NETs promoted the expression of Th2-like cytokines, airway eosinophil infiltration and airway hyperresponsiveness (AHR) [152]. NETs may not only have pathogenic effects but may also exert beneficial effects in human and experimental models of asthma [9]. NETs can capture and/or kill bacteria [15,182], viruses [161,167,183], and fungi [162,172,184]. NETs can also promote the resolution of inflammation by degrading cytokines and chemokines [168,169]. NETs inhibit GM-CSF/IL-4-induced dendritic cell (DC) differentiation [173]. In experimental models of asthma, MMP-9 reduced ROS accumulation and DNA damage [185].

Figure 3 schematically summarizes the proinflammatory and anti-inflammatory effects of NETs in humans and experimental models of inflammation.

## 7. Physiologic States That Influence NET Biology

Neutrophil biology is influenced by several variables in the host, such as age, sex, and circadian rhythms [9,186]. For instance, neutrophils expressing an immature phenotype are increased in young males compared to females [187,188]. Interestingly, women have an increased capacity to form NETosis [187,188]. Moreover, neutrophil phenotype is altered during pregnancy as estrogen levels increase [189]. In both males and females, neutrophils are diurnally replenished and there is a distinct circadian regulation of neutrophil aging [190,191]. Neutrophil aging is modulated in part by the microbiome through the engagement of TLR4 by LPS [53], a potent stimulus for NET formation [18,72,80,138]. NET formation induced by inflammatory stimuli is impaired in older individuals compared to young adults [192]. Although age [193,194] and sex [194,195,196] can affect asthma prevalence and/or severity, the relationship between these factors and NET formation remains unexplored. From a practical point of view, these findings suggest that studies on the role of NETs in asthma should take into account age, sex, and the timing of blood and other tissue sampling.

## 8. NET Biomarkers

In recent decades, strong efforts have been made to identify reliable biomarkers of different phenotypes of asthma, such as T2-high and T2-low asthma [197,198,199]. In particular, biomarkers to assess non-type 2 asthma still represent an unmet need [200]. There is potential for NETs to become novel biomarkers of asthma progression or severity as well as therapeutic targets. A variety of different analytical approaches (e.g., double-strand DNA (dsDNA), myeloperoxidase-DNA (MPO-DNA), and citrullinated histone H3 (CitH3)) have been used as a surrogate measurement of NETs in asthma [27,103,108,139,201,202,203]. Putative NET biomarkers have been found in different sites/biological fluids of patients with asthma. The presence of DNA was initially detected in bronchial biopsies of patients with asthma [154]. Additionally, dsDNA has been found in the sputum [27,108], nasal lavage [139] and BAL of patients with asthma [179,202]. The measurement of dsDNA has been widely used as a NET biomarker [27,108,139]. However, the quantitative determination of circulating DNA does not necessarily reflect the concentration of NETs in vivo. For instance, DNA complexes may result from other forms of cell death related to neutrophilic inflammation [201,204]. MPO-DNA has been proposed as a circulating biomarker of NETs in severe asthma [202]. However, this assay is error-prone and does not offer complete information about NETs in vivo [203]. We demonstrated that plasma concentrations of two NET components, CitH3 and dsDNA, were increased in asthma patients compared to healthy donors and inversely correlated with the percent decrease in FEV_1_/FVC [103].

It should be emphasized that no validated assay available today has proven to be clinically useful. Indeed, there is a lack of standardization in the measurement of putative NET biomarkers as well as problems related to sensitivity and specificity. Thus, there are no currently accepted values for these parameters in relation to NETs in asthma or in other diseases. In conclusion, the evaluation of different NET biomarkers in different phenotypes of asthma (alone or in combination with other parameters) and during acute asthma exacerbations requires further study.

## 9. Future Perspective: NET Inhibitors

Inhibition of NET formation might represent a possible target for treating certain endophenotypes of asthma. A variety of inhibitors that prevent NET formation, as well as molecules that degrade NETs, are currently under investigation for the treatment of cancer and inflammatory diseases [19]. Recombinant human protease inhibitors (e.g., NE inhibitors) and DNase can neutralize and degrade NET-derived DNA and mediators, reducing their proinflammatory effects [205]. DNase, degrading chromatin within the NETs, is a promising strategy to interfere with NET formation and activity [52,206]. Anti-histone antibodies also have some proven efficacy in treating autoimmune disorders [207]. PAD4 appears to be an interesting target in several mouse models to reduce NET-mediated inflammation. PAD inhibitor Cl-amidine also inhibits histone citrullination, which is a pivotal step in NETosis [207]. However, NETosis may not always depend on PAD4 [208] and PAD4 inhibitor efficacy may have species-specific variations [209].

The blockade of NE has also been shown to inhibit NET-induced disruption of endothelial cell–cell integrity [210]. Other molecules are being examined for their potential to inhibit NETosis. The inhibition of NADPH oxidase is effective in preventing suicidal NETosis in vitro. However, studies on experimental murine models of SLE [170] and gout [168], both of which lack NADPH oxidase, have shown more severe disease. Similarly, patients with chronic granulomatous disease, who have defects in NADPH oxidase, have a greater incidence of autoimmune disorders [211].

It should be pointed out that although these pharmacologic strategies have advantages in the treatment of possible NET-mediated diseases, complete NET inhibition in animal models increases susceptibility to infections and decreases neutrophil functions involved in innate immunity [212,213]. There is also evidence that ROS signaling and NET presence are important to modulate inflammation; whereas, excessive NETs can actually be responsible for disease. Several compounds have been tested for their ability to limit NETosis without interfering with ROS production in vitro. An example of such a compound is tetrahydroisoquinoline [214], which inhibits both spontaneous and PMA-induced NETosis in neutrophils from SLE patients. Further studies and comprehension of the regulation and balance of NET formation, inhibition, and degradation using NET inhibitors will be mandatory to avoid compromising the patient’s immune system [205].

## 10. Extracellular Traps (ETs) from Other Immune Cells

Compelling evidence indicates that several immune cells (i.e., eosinophils, mast cells, macrophages, and basophils) and their mediators contribute to airway inflammation in different asthma phenotypes [215,216,217]. The first description of extracellular traps (ETs) from neutrophils occurred in 2004 [15]. Since then, ETs have been identified in several other cells of the innate immune system, including eosinophils [218,219], basophils [66,220,221], mast cells [222,223,224,225,226,227], and macrophages [228,229,230]. Therefore, the term ETosis has been coined as a conventional/generic term for immune cells releasing ETs. Increasing evidence suggests that a single cell type can display multiple ET formation mechanisms and that different immune cells can display similar ET formation mechanisms [97,231,232,233].

Eosinophil extracellular traps (EETs) are composed of a meshwork of DNA and eosinophil granule proteins, such as major basic protein (MBP) and eosinophil cationic protein (ECP) [201,219]. Notably, the release of mtDNA by eosinophils occurs rapidly in a catapult-like manner [182]. Eosinophils and their powerful mediators play a key role in the pathogenesis of allergic and eosinophilic asthma [234]. EETs can be released by activated eosinophils contributing to airway inflammation in severe asthma [218]. Vital and suicide EETs have also been associated with other allergic eosinophilic diseases, including chronic rhinosinusitis with nasal polyps and eosinophilic esophagitis [154,235,236,237,238].

Macrophage extracellular traps (METs) contain DNA (mtDNA and/or nuclear DNA) but also CitH3, MPO and lysozyme [228,239]. Macrophages are the predominant immune cells in the human lung and release a plethora of proinflammatory mediators that are involved in asthma. [240,241]. Several infectious and non-infectious stimuli can induce the release of METs [220,229,230].

Mast cell extracellular traps (MCETs) are composed of nuclear DNA, histones, tryptase, and antimicrobial peptides such as cathelicidins [224,225,227,242]. Mast cells and their mediators play a key role in the pathobiology of different asthma phenotypes [215,243,244]. MCETs are released from mast cells in response to a variety of immunologic (e.g., anti-IgE, substance P) [245] and chemical stimuli (e.g., H_2_O_2_, PMA) [242], and to various pathogens including bacteria, fungi, viruses and protozoa [222,223,224,225,226,227].

Even though basophils account for ≅1% of circulating leukocytes, these cells can play critical roles in the activation of type 2 immune responses in allergic asthma [6,52,246]. Basophil extracellular traps (BETs) are released within a few minutes of stimulation by IgE, chemokines, cytokines, TLRs, monosodium urate crystals and lipid mediators [220,221]. BETs are composed of mtDNA and are formed by a mtROS-dependent and NADPH oxidase-independent mechanism [66]. Further studies in experimental models and using human biological materials will be necessary to verify the involvement and the effects of different ETs in asthma pathobiology.

## 11. Conclusions and Future Perspectives

Asthma is a complex and heterogeneous disease with variable clinical manifestations [247,248,249]. The role of NETs or possibly other forms of ETosis (e.g., EETs, METs, MCETs, BETs) in different asthma endophenotypes is still largely unknown. The discovery of NETs has changed the paradigm of neutrophil-mediated innate immunity. Although NET formation is now recognized as a powerful antimicrobial defense system [250], its dysregulation may contribute to chronic airway inflammation. There is experimental evidence in preclinical models that NET formation is also implicated in asthma exacerbations and that a unique population of SiglecF^+^ PMNs is involved [153]. The role of Siglec8^+^ neutrophils and NET formation should also be investigated during asthma exacerbations.

NETopathic inflammation can be avoided or reversed by inhibiting NET formation, blocking the granular proteins decorating NETs, or clearing NETs that have already been released. Inhibition of NET formation has started to be investigated in experimental models of asthma. Vargas et al. found that glucocorticoids decreased NET formation in vitro and in vivo in the lungs of asthmatic horses [251]. Inhibition of NE, a NET component, can attenuate rhinovirus-induced airway hyperreactivity in a mouse model of asthma [139]. Patients with asthma on inhaled glucocorticoid (ICS) treatment showed lower circulating NET levels compared to patients who did not or just occasionally used ICS [252]. Future studies should investigate whether pharmacological or biological therapies for asthma could modify the expression of NETs and their circulating biomarkers in vivo.

Despite the growing evidence, important questions remain unanswered. Table 1 summarizes some limitations in studying NETs in asthma. It remains unclear whether NETs are a *double-edged* sword in asthma. It is not known whether or how NET formation contributes to the pathogenesis of asthma or whether stabilizing this process could be an effective therapeutic strategy. It is also crucial to consider that NETs, and presumably other forms of ET, play an important role in host defense. Therefore, therapeutic intervention should be cautiously targeted to modulate the potential of NETs toward maintaining homeostasis rather than attempting to neutralize them completely. In conclusion, we anticipate that a better knowledge of the biochemical and immunological features of NETopathic inflammation could lead to the development of innovative and personalized therapies for specific asthma endophenotypes.

## Figures and Tables

**Table 1:** Limitations in studying NETs in asthma.

•Most if not all studies on the role of NETs in asthma have been performed on bulk analysis of neutrophils. Human neutrophils are highly heterogeneous [45,46,47,48], and different subsets of neutrophils could produce different forms of NETs.
•We do not know the role of neutrophil subsets and their specific mediators, including NETs, in different phenotypes of asthma.
•We do not know whether subsets of neutrophils make different NETs.
•We do not know whether NETs in different phases (early vs. late) or phenotypes of asthma (T2-high vs. T2-low) are proinflammatory or anti-inflammatory.
•In human studies, most experimental approaches are limited to ex vivo analyses of NET formation by blood neutrophils and basic correlations with clinical outcomes. However, these approaches do not provide insights into the underlying causes of disease.
•Approximately 25% of neutrophils release vital NETs [74]. We do not know the role of non-lytic and lytic NETs in asthma.
•Primary human neutrophils cannot undergo transfection and it is difficult to specifically inhibit pathways that lead to NET formation in vivo.
•Several immune cells, such as eosinophils, mast cells, macrophages, and basophils can release extracellular DNA traps.
•In vivo identification of NETs requires the colocalization of at least three molecules: extracellular DNA, histones, and neutrophil elastase.
•We do not know whether allergen immunotherapy modulates NET formation in vivo.

## Data Availability

Not applicable.

## Abbreviations

ACO, asthma-COPD overlap; aggNET, aggregated NET; AIT, allergen immunotherapy; alum, aluminum hydroxide; AMP, antimicrobial peptide; ANGPT1, angiopoietin-1; APC, antigen-presenting cell; ATP, adenosine triphosphate; BAL, bronchoalveolar lavage; BEC, bronchial epithelial cell; BET, basophil extracellular trap; CitH3, citrullinated histone H3; COPD, chronic obstructive pulmonary disease; CysLT, cysteinyl leukotriene; DAMP, Damage-associated molecular pattern; DC, dendritic cell; DEP, diesel exhaust particle; dsDNA, double strand DNA; *E. coli*, *Escherichia coli*; ECM, extracellular matrix; ECP, eosinophil cationic protein; eDNA, extracellular DNA; EET, eosinophil extracellular trap; ET, extracellular trap; FEV_1_, forced expired volume; fMLP, N-formyl peptide; FVC, forced vital capacity; HDM, house dust mite; HMBG1, high mobility group box 1 protein; ICS, inhaled glucocorticoid; ILC2, innate lymphoid cells type 2; LPS, lipopolysaccharide; MBP, major basic protein; MCET, mast cell extracellular trap; MET, macrophage extracellular trap; MMP9, matrix metallopeptidase 9; mtDNA, mitochondrial DNA; MPLA, monophosphoryl-lipid A; MPO, myeloperoxidase; MPO-DNA, myeloperoxidase-DNA; mROS, mitochondrial ROS; NADPH, nicotinamide adenine dinucleotide phosphate; NE, neutrophils elastase; NET, neutrophil extracellular trap; PAD, peptidyl arginine deiminase; PKC, protein kinase C; PMA, phorbol 12-myristate 13-acetate; PMN, polymorphonuclear leukocyte; PR3, proteinase 3; PTX3, pentraxin 3; Th2, T2 helper cell; ROS, reactive oxygen species; *S. aureus*, *Staphylococcus aureus*; SLE, systemic lupus erythematosus, TSLP, thymic stromal lymphopoietin; VEGF, vascular endothelial growth factor.
